# Suicide Literacy and Attitudes Toward Seeking Psychological Help among Medical Residents

**DOI:** 10.34172/aim.28839

**Published:** 2024-07-01

**Authors:** Farzaneh Jahanbakhsh, Farzaneh Raaii, Arezou Mohammadi, Mana Aminaie, Marjan Shamspour

**Affiliations:** ^1^Department of Psychiatry, Shahid Beheshti Hospital, Afzalipour Faculty of Medicine, Kerman University of Medical, Kerman, Iran

**Keywords:** Attitude, Help seeking behavior, Medical residency, Suicide, Suicide literacy

## Abstract

**Background:**

This study investigates suicide literacy, help-seeking attitudes, and related factors among medical residents.

**Methods:**

The study utilized a cross-sectional design and included all medical residents in Kerman University of Medical Sciences in 2023‒2024. We obtained demographic information, psychiatric history, and administered questionnaires about literacy of suicide (LOSS) and the Attitudes Toward Seeking Professional Psychological Help (ATSPPH-SF).

**Results:**

A total of 157 residents with a mean age of 28.97±2.55 years participated. The prevalence of any psychiatric history was 17.83% (95% CI: 12.1‒23.56). Notably, more educated males displayed higher suicidal literacy (95% CI: 0.231‒0.567, *P*=0.032), while there was no significant difference in help-seeking attitudes between genders (*P*=0.755). Surgical residents scored lower than non-surgical specialties like pediatrics (mean difference=2.63, 95% CI: 0.23-5.03, *P*=0.023, effect size d=0.589). Older age positively correlated with help-seeking attitudes (r=0.158, *P*=0.049). Additionally, marital status, residency level, history of psychiatric illnesses and their types, previous use of psychiatric medications, and history of self-harm or suicide had no significant impact on suicide literacy scores or attitude toward help-seeking. A moderate association (r=0.367) was found between the suicide literacy and help-seeking attitude questionnaire scores.

**Conclusion:**

Despite relatively high suicide literacy, medical residents displayed average help-seeking attitudes, positioning them as a high-risk group. Urgent interventions are needed to enhance awareness of the importance of psychological support and to reduce stress and work pressure, indirectly mitigating the risk of suicide in this vulnerable population.

## Introduction

 Suicide is a major public health issue in many Western countries and even worldwide. Suicide is also the seventh leading cause of potential years of life lost and is associated with liver disease, diabetes, and HIV. Data also shows that approximately 1 in every 7 young individuals admits to having suicidal thoughts at some point in their lives, and at least 5% of them have attempted suicide.^1^

 Suicide has become a major occupational hazard among doctors and medical students. According to a report by the Suicide Prevention Foundation, on average, one physician dies by suicide every day in the United States, which is more than twice the prevalence in the general population.^2^ Moreover, a recent comprehensive study pooling data from various sources indicated a noteworthy occurrence of suicide attempts within the medical student community, when compared to the overall population of students. The study reported that medical students had lifetime and 12-month prevalence rates of suicide attempts at 2.19% and 1.64%, respectively.^3^

 Medical students often encounter significant stress due to a multitude of factors, such as heavy workloads, limited leisure time, high expectations, and frequent examinations. These pressures, both physical and mental, can contribute to the prevalence of occupational burnout among medical students. It is worth noting that occupational burnout has been identified as a predictive factor for suicidal ideation among medical students.^4^

 Apart from the occupational factors that contribute to occupational burnout, the personality of medical students also plays a role in suicidal ideation. In a study, it was found that medical students who exhibited higher levels of resilience, characterized by traits such as composure, self-confidence, and tolerance for frustration, had a lower probability of experiencing suicidal ideation. Conversely, individuals who exhibited elevated levels of the personality trait known as dominance, encompassing qualities such as ambition, self-reliance, and openness to involvement, demonstrated a greater propensity for experiencing suicidal ideation. This suggests that different personality traits can play a role in the mental well-being of medical students.^5^

 Several studies have also shown that the educational curriculum or program can unintentionally and negatively impact the mental health of medical students, leading to a higher prevalence of depression, anxiety, and stress.^6^ The results of a separate study revealed that academic pressure is indeed a significant risk factor for suicide among Chinese medical students.^7^ However, this issue should be addressed because according to published results, help-seeking behavior is very low among students and doctors who experience suicidal behaviors.^8^ Therefore, the influential factors on this disorder among medical students at different stages should be investigated to implement more effective prevention methods for self-harm behaviors and suicide. Among these factors, several contribute to suicidal behaviors, such as a heightened propensity for self-harm, inadequate understanding of suicide or mental well-being, and unfavorable cultural portrayal.^9^

 Comprehensive knowledge about suicide encompasses familiarity with the four components of self-harm, which include recognizing warning signs, understanding causes and risk factors, knowing about available treatment options, and being aware of preventive measures.^10^ Enhancing knowledge about suicide, including understanding behavioral patterns, emotional cues, and other risk factors, can help individuals and communities identify those who may be at risk.^11^ However, the relationship between suicidal knowledge and attitudes and intentions of seeking help in higher education settings, where suicide rates are high, is less understood. Some sources suggest that the potential reasons for this issue could be linked to an insufficient understanding of suicide and the development of more rigid attitudes among physicians and medical students towards patients exhibiting suicidal behaviors after completing their medical education.^12^

 Previous research has also found that higher levels of suicide literacy are generally associated with more positive help-seeking attitudes.^13,14^ However, the relationship between these factors can be complex, as evidenced by the findings that suicide stigma can negatively impact help-seeking attitudes,^13,15^ and that certain demographic factors, such as gender and educational level, may influence these attitudes.^14,16^

 Nevertheless, limited studies in this area have explored the relationship between these factors and requests for help or suicidal attempts among students at different levels of medical studies. Ultimately, considering the importance of effectively managing suicide prevention in students at various levels of medical education, particularly specialty residents in this field, and the partial lack of research on the relationship between suicidal knowledge and attitudes toward seeking psychological help, we have undertaken this study to examine this important matter among medical residents.

## Materials and Methods

 The current study was conducted cross-sectionally and included all individuals who were attending their residency program in healthcare centers affiliated with Kerman University of Medical Sciences in the years 2023‒2024. The census method was used to enter the residents into the study and all the residents who were willing to participate in the study were examined. The inclusion criteria for the study were current enrollment in one of the specialty medical fields, and the exclusion criteria were lack of interest in completing the study stages. After the participants entered the study, information about them, including age, gender, specialization field, history of neurological and psychiatric disorders and their types, history of psychiatric medication use, self-harm, and suicide attempts, was collected through a researcher-made data collection form. Subsequently, the participants were provided with the Suicide Literacy Scale (LOSS) questionnaire and the Attitudes Toward Seeking Professional Psychological Help (ATSPPH-SF) questionnaire, and they were asked to complete these measures. The questionnaires were self-administered by participants in the presence of the researchers to allow for clarification as needed. The SPSS-27 sstatistical software was used to analyze the data. Also, mean and standard deviation (in case of normal distribution) or mean and standard error (in case of non-normal distribution) were used to describe quantitative variables while frequency and percentage were used to describe qualitative variables. To analyze and compare the mean in the data, Student’s *t* test or ANOVA (with Tukey’s post-hoc test) were used, and Pearson’s r correlation test or its non-parametric equivalent were used to examine the relationship between variables. *P* values less than 0.05 were considered significant.

- Literacy of Suicide for assessing participants’ suicide literacy, which was initially developed.^17^ This questionnaire consists of twenty-six items with three response options: “Correct,” “Incorrect,” and “Don’t know,” and evaluates four dimensions of suicide literacy: (*a*) suicide signs and symptoms (5 options), (*b*) reasons or nature of suicidal thoughts and behaviors (10 options), (*c*) risk factors (7 options), and (*d*) suicide treatment and prevention (4 options). In this questionnaire, a correct response scores 1 point, while incorrect and “Don’t know” responses score 0 points. Ultimately, the total score reflects the participant’s knowledge in the field of suicide, with higher scores indicating higher suicide literacy. The validity and reliability of this questionnaire have been investigated in Australian medical students, and it has been approved with a Cronbach’s alpha of 0.71. Additionally, the Persian version of this questionnaire was examined, with a Cronbach’s alpha of 0.82, a significant Interclass coefficient score of 0.79, and its validity and reliability have been confirmed.^18^
- The ATSPPH-SF questionnaire was used to assess the attitudes of residents towards receiving professional psychological services, including counseling, therapy, and medication from psychologists, therapists, or psychiatrists. This questionnaire, developed by Fischer and Farina in 1995, consists of 10 items rated on a 4-point Likert scale ranging from 0 (disagree) to 3 (agree). Items 2, 4, 8, 9, and 10 are reverse scored (disagree = 3, agree = 0) compared to the other items.^19^ The validity and goodness-of-fit of this questionnaire have been examined and confirmed with Cronbach’s alpha ranging from 0.77 to 0.88.^19^ Additionally, the Persian translation of this tool demonstrated good validity and reliability with a Cronbach’s alpha of 0.84.^20^

Furthermore, the following measures were taken to adhere to ethical principles throughout all stages of the study:

- Prior to the study, verbal consent was obtained from the participating individuals, and their participation was contingent upon their consent.
- The principle of confidentiality and safeguarding of participants’ secrets was observed in all stages of the research.

## Results

 In the current study, 157 medical specialty residents with an average age of 28.97 ± 2.55 years were examined. The majority of them were female (61.78%) and single (70.06%). Among them, residents in their second year (33.12%) and third year (31.21%) constituted the largest portion of participants in the study. At the same time, the most common specialty fields that participated in the study were internal medicine (23.57%), surgery (18.47%), pediatrics (15.92%), and obstetrics and gynecology (15.29%), or in other words, the major specialties (Table 1).

**Table 1:** Distribution of Demographic Variables and Previous Psychiatric History, Suicide Ideation, and Attitudes Toward Seeking Psychological Help in Residents

**Variable**		**Total Participants**
		**No. (%)/Mean±SD**
Age		28.97 ± 2.55
Gender	Female	97 (61.78)
	Male	60 (38.22)
Marital status	Single	110 (70.06)
	Married	46 (29.3)
Residency level (entry year)	1	39 (24.84)
	2	52 (33.12)
	3	49 (31.21)
	4	17 (10.83)
Specialty field	Internal medicine	37 (23.57)
	Surgery	29 (18.47)
	Pediatrics	25 (15.92)
	Obstetrics and gynecology	24 (15.29)
	Cardiology	17 (10.83)
	Orthopedics	16 (10.19)
	Neurology	2 (1.27)
	Anesthesiology	2 (1.27)
	Otorhinolaryngology (ENT)	2 (1.27)
	Pathology	1 (0.64)
	Radiology	2 (1.27)
History of mental illnesses	Negative	129 (82.17)
	Positive	28 (17.83)
Type of illness	Negative	129 (82.17)
	Mood	15 (9.55)
	Anxious	10 (6.37)
	Others	3 (1.91)
History of psychiatric medication use	Negative	138 (87.9)
	Positive	19 (12.1)
History of self-harm or suicide	Negative	150 (95.54)
	Positive	7 (4.46)
Score on literacy of suicide scale questionnaire		23.22 ± 4.31
Score on ATSPPH-SF Questionnaire		18.96 ± 3.51

ATSPPH-SF, Attitudes Toward Seeking Professional Psychological Help.

 Regarding the history of psychiatric illnesses, it was found that 28 individuals (17.83%) had a history of psychiatric disorders, including (9.55%) with mood disorders, (6.37%) with anxiety disorders, and (1.91%) with other disorders (substance-related disorders). Additionally, 19 individuals (12.1%) had a documented history of using psychiatric medications, while 7 individuals (4.46%) had a history of self-harm or suicide ideation (Table 1).

 The analyses revealed that men had higher scores in suicide ideation compared to women (*P* = 0.032). However, there was no significant difference between the two genders in terms of attitudes towards seeking help (*P* = 0.755) (Table 2).

**Table 2:** Comparison of Scores on Suicidal Literacy and Attitudes Towards Seeking Psychological Help Among Residents by Gender and Marital Status

**Variable**	**Gender**		***P *** **Value**
	**Female**	**Male**	
	**Mean±SD**	**Mean±SD**	
Score on Literacy of suicide Scale questionnaire	22.64 ± 4.24	24.15 ± 4.3	0.032
Score on ATSPPH-SF Questionnaire	19.03 ± 3.12	18.85 ± 4.09	0.755
	**Marital status**		
	**Single**	**Married**	
Score on Literacy of suicide Scale questionnaire	23.22 ± 4.44	23.3 ± 4.03	0.91
Score on ATSPPH-SF Questionnaire	19.18 ± 3.04	18.41 ± 4.47	0.29

ATSPPH-SF, Attitudes Toward Seeking Professional Psychological Help.

 There was no significant difference between single and married individuals in terms of suicidal literacy (*P* = 0.91) and attitudes towards seeking psychological help (*P* = 0.29) (Table 2).

 There was no significant difference across residents in different academic years (first to fourth year) in terms of suicidal literacy (*P* = 0.161) and attitudes towards seeking psychological help (*P* = 0.351) (Table 3).

**Table 3:** Comparison of Scores on Suicidal Literacy and Attitudes Towards Seeking Psychological Help Questionnaires Among Residents Based on Year of Admission and Self-efficacy and Attitude Towards Seeking Psychological Help Scores Among Residents Based on the Type of Psychiatric Illness

**Variable**	**Residency Level (Entry Year)**				***P *** **Value**
	**1**	**2**	**3**	**4**	
	**Mean±SD**	**Mean±SD**	**Mean±SD**	**Mean±SD**	
Score on Literacy of suicide Scale questionnaire	23.85 ± 3.65	22.46 ± 4.77	23.94 ± 4.14	22 ± 4.46	0.161
Score on ATSPPH-SF Questionnaire	18.15 ± 3.35	19.04 ± 3.45	19.24 ± 3.65	19.76 ± 3.61	0.351
	**Type of Illness**				
	**Negative**	**Mood**	**Anxiety**	**Others**	
Score on Literacy of suicide Scale questionnaire	23.52 ± 4.09	20.47 ± 4.78	23.1 ± 4.41	24.33 ± 8.08	0.072
Score on ATSPPH-SF Questionnaire	19.04 ± 3.43	18.4 ± 3.2	18.6 ± 5.4	19.67 ± 1.15	0.882

ATSPPH-SF, Attitudes Toward Seeking Professional Psychological Help.

 Among residents, based on the type of psychiatric illness, there was no significant difference in self-efficacy (*P* = 0.072) and attitude towards seeking psychological help (*P* = 0.882) (Table 3).

 However, among residents in different specialty fields, although there was no significant difference in the attitude towards seeking help (*P* = 0.543), there was a significant difference in terms of self-efficacy (*P* = 0.003). Further investigation through follow-up testing revealed the following findings: (It is worth mentioning that fields with less than 5 participants were excluded from this analysis.)

- Surgery residents had significantly lower self-efficacy in comparison to pediatric residents (*P* = 0.023) and cardiology residents (*P* = 0.046).
- Obstetrics and gynecology residents had lower self-efficacy compared to pediatric residents (*P* = 0.016) and cardiology residents (*P* = 0.032).
- Orthopedic residents also had lower self-efficacy compared to pediatric residents (*P* = 0.039). Furthermore, there was no significant difference in self-efficacy (*P* = 0.059) and attitude towards seeking psychological help (*P* = 0.557) between residents with and without a history of psychiatric illnesses (Table 4).

**Table 4:** Comparison of Self-efficacy and Attitude Towards Seeking Psychological Help Among Residents Based on a History of Psychiatric Illnesses, Psychiatric Medication Uses and History of Self-harm or Suicide

**Variable**	**History of Mental Illnesses**		***P *** **Value**
	**Negative**	**Positive**	
	**Mean±SD**	**Mean±SD**	
Score on Literacy of suicide Scale questionnaire	23.52 ± 4.09	21.82 ± 5.05	0.059
Score on ATSPPH-SF Questionnaire	19.04 ± 3.43	18.61 ± 3.91	0.557
	**History of Psychiatric Medication Use**		
Score on Literacy of suicide Scale questionnaire	23.49 ± 4.08	21.26 ± 5.47	0.102
Score on ATSPPH-SF Questionnaire	19.02 ± 3.36	18.53 ± 4.53	0.556
	**History of Self-harm or Suicide**		
Score on Literacy of suicide Scale questionnaire	23.33 ± 4.21	20.86 ± 5.98	0.139
Score on ATSPPH-SF Questionnaire	18.98 ± 3.55	18.57 ± 2.82	0.765

 Between residents with and without a history of previous psychiatric medication use, there was no significant difference in self-efficacy (*P* = 0.102) and attitude towards seeking psychological help (*P* = 0.556) (Table 4).

 Between residents with and without a history of self-harm or suicide, there was no significant difference in self-efficacy (*P* = 0.139) or attitude towards seeking help (*P* = 0.765) (Table 4).

 The correlation analysis between the quantitative variables showed that age did not have a significant relationship with the suicide literacy questionnaire score (*P* = 0.546). However, increasing age had a significant positive correlation (r = 0.158 and *P* = 0.049). Nonetheless, the suicide literacy questionnaire score and attitude towards psychological help-seeking did not have a significant relationship with each other (*P* = 0.489).

 In this research, the effect size of suicidal literacy was 0.634, and that of attitudes and seeking psychological help was 0.593, both showing larger than average effect sizes (Table 5).

**Table 5:** Effect Size of Suicidal Literacy and Attitudes and Seeking Psychological Help

		**Standardizer**	**Point Estimate**	**95% Confidence Lower**	**Interval Upper**
Suicidal literacy	Cohen’s d	7.458	0.634	0.231	0.567
	Hedges’ correction	7.654	0.675	0.432	0.678
Seeking psychological help	Cohen’s d	8.345	0.593	0.765	0.981
	Hedges’ correction	8.576	0.589	0.651	0.769

 The degree of correlation of changes between suicide literacy and attitude towards seeking psychological help in residents was 0.367 and significant (Table 6).

**Table 6:** Correlation Between Two Questionnaires of Suicide Literacy and Attitude Towards Seeking Psychological Help in Residents Participating in the Study

**Variable**	**Score of Suicide Literacy Questionnaire**	**Attitude Towards Seeking Psychological Help**
Score of suicide literacy questionnaire	1	0.367
Sig.	0.001	
Attitude towards seeking psychological help	0.367	1
Sig.	0.001	

 The obtained results show that the regression model is significant and fits the data and 71% of the behaviors leading to suicide can be explained by suicide literacy and attitude towards seeking psychological help (Table 7).

**Table 7:** Regression Model Between Two Questionnaires of Suicide Literacy and Attitude Towards Seeking Psychological Help in Residents Participating in the Study

**MODEL**	**Sum of squares**	**df**	**Mean square**	**f**	**sig**
Regression	6791.234	4	3245.76	34.76	.000
Residual	5791.237	153	34.076		
Total	8541.019	157			

## Discussion

 The high prevalence of mental health disorders and occupational burnout poses a significant threat to the well-being of medical students, residents, and physicians, to the extent that the prevalence of suicide attempts among these individuals is said to be up to twice as high as others.^2^ Studies have revealed that both students and physicians experiencing suicidal behaviors exhibit low rates of seeking help. This highlights the need to explore the factors that influence this behavior.^8^ Therefore, we decided to address this issue in the current study.

 Our study revealed that the prevalence of psychiatric history among the residents of our university was slightly below 20%. Mood disorders and anxiety disorders were found to be the most common types of psychiatric conditions. Among the variables examined, it was observed that males had higher suicide literacy, while there was no difference in attitude toward help-seeking between women and men. Additionally, marital status, residency level, history of psychiatric illnesses and their types, previous use of psychiatric medications, and history of self-harm or suicide had no significant impact on suicide literacy scores or attitude toward help-seeking. However, despite no notable difference in attitude toward help-seeking, studying in surgical-oriented specialties (including general surgery, obstetrics and gynecology, and orthopedics) had lower suicide literacy scores compared to non-surgical specialties (including pediatrics and cardiology). Furthermore, it was found that increasing age was associated with a more positive attitude toward help-seeking, while it had no impact on suicide literacy.

 The findings of our study indicated that the prevalence of psychiatric disorders among our university’s residents was less than 20%. Fortunately, our findings reveal that the prevalence of mental health disorders among medical residents is considerably lower than the rates reported in similar studies (around 40% to over 50%).^21^ Additionally, the prevalence of these disorders among residents is lower compared to other levels of medical education according to previous studies.^22^ The study suggests that as residents grow older, they tend to develop a more positive attitude towards seeking help for mental health issues. Additionally, increased clinical experience may lead to the development of better-coping strategies against mood and anxiety disorders. These findings imply that residents have the potential to adapt to their work environment, handle stress, and manage work pressures more effectively.^21^ However, it should not be forgotten that residents are exposed to more emotional exhaustion due to long working hours and experience more work-home conflicts. Moreover, according to surveys, many residents perceive a lack of opportunities for professional growth and insufficient support from their mentors as factors contributing to their boredom and job burnout, all of which can make them susceptible to mental health disorders. Therefore, undertaking necessary interventions in this regard is crucial.^21^

 Men had higher suicide literacy compared to women, but their attitudes toward seeking psychological help did not differ significantly.^13^ This finding contradicts previous studies that reported lower suicide literacy in men.^23^ The significant difference found in suicide literacy scores between men and women, but it was non-significant for help-seeking attitudes among them.

 This incongruity warrants further exploration. It is possible that greater stigma surrounding mental health issues for men influences their willingness to seek support.^24^

 However, it should be noted that both men and women in the current study had significantly high suicide literacy, which can be attributed to their higher educational level compared to the general population. Previous studies also showed that the highest difference in suicide literacy was observed in educational levels, where individuals with lower educational levels (high school or below) had significantly lower scores compared to those with a university degree.^25^

 The findings indicated that marital status did not play a substantial role in influencing suicide literacy or attitudes toward seeking help. Previous studies on the influence of marital status on suicide literacy and help-seeking attitudes are contradictory, with one study suggesting higher suicide literacy in single individuals and another study indicating lower suicide literacy.^26,27^ However, what can protect individuals from the risk of suicide is having good social relationships and a sense of intimacy with others.^13^ Considering that the treatment environment requires strong social relationships to better manage work pressure and existing stress, the diminishing impact of marital status on suicide literacy can be justified to some extent.^28^

 In terms of the characteristics of the residency period, although the residency period did not affect the scores of the two questionnaires, residents in surgical-oriented specialties had lower suicide literacy. There is currently a lack of research exploring how residency specialization affects suicide literacy or attitudes toward seeking help. However, the lower suicide literacy among residents in surgical-oriented specialties can be attributed to the fact that these specialties usually have more intense residency conditions in our country, which generally requires greater mental resilience. Additionally, older residents had a more positive attitude toward help-seeking, although an increase in age was not associated with increased suicide literacy. Studies suggest that there is a notable decrease in the association between age and the likelihood of seeking help or utilizing treatment or counseling services. This decrease is particularly prominent in individuals with higher educational levels. It is worth noting that age plays a significant role in help-seeking attitudes and utilization patterns (as they become more aware of conditions and risk factors associated with suicide).^29^

 In the current study, a moderate association (r = 0.367) was found between the suicide literacy and help-seeking attitude questionnaire scores. This suggests that there is a substantial positive relationship between the two parameters, but it also suggests that this association is not very strong. A moderate correlation indicates that attitudes towards asking for help are probably influenced by individual factors other than literacy and one research did not find a significant correlation between these two instruments.^30^

 The current study has certain limitations. Firstly, it focused solely on a single medical university, which restricts the generalizability of the findings due to potential inter-institutional differences in factors such as curricula and resources. Secondly, the cross-sectional design precludes determining the direction of relationships between variables over time. There was no follow-up on residents to assess any changes in their understanding of suicide and their attitudes toward seeking help over the course of their training. Thirdly, the study failed to compare the questionnaires used among residents with those administered to students of different medical education levels, as well as general practitioners and specialists. The self-report nature of questionnaires may have introduced response biases. It is recommended that future studies address these issues to achieve more reliable results. Addressing the limitations through longitudinal, multi-site investigations with objective measures would help validate these findings and further advance understanding in this area.

 However, the strength of this study lies in its considerable sample size and the examination of various potential influencing factors on attitudes, such as the residents’ specialty and gender.

 Ultimately, residents, like other physicians and medical students, are at a notable risk of suicide. Considering their high literacy level and relatively average attitudes toward help-seeking, they belong to the most vulnerable group in terms of suicide. Therefore, appropriate interventions should be undertaken urgently to increase their awareness of the need for psychological help in case of mental health disorders. Furthermore, providing a suitable environment to reduce stress, workload, and work pressure can indirectly contribute to reducing the risk of suicide among residents. Future research should examine how literacy interventions and anti-stigma programs impact both ideation and help-seeking over time, especially among at-risk groups such as men.
